# Response scale selection in adult pain measures: results from a literature review

**DOI:** 10.1186/s41687-018-0053-6

**Published:** 2018-09-06

**Authors:** Shima Safikhani, Katharine S. Gries, Jeremiah J. Trudeau, David Reasner, Katja Rüdell, Stephen Joel Coons, Elizabeth Nicole Bush, Jennifer Hanlon, Lucy Abraham, Margaret Vernon

**Affiliations:** 1 Evidera, 7101 Wisconsin Ave., Suite 1400, Bethesda, MD 20814 USA; 2 Janssen Global Services LLC, 700 Route US 202, Raritan, NJ 08869 USA; 3 Ironwood Pharmaceuticals, 301 Binney Street, Cambridge, MA 02142 USA; 4 Pfizer LTD, Outcomes & Evidence, Global Health & Value, Pfizer Ltd, Tadworth, Surrey UK; 5 Patient-Reported Outcome Consortium, Critical Path Institute, 1730 E River Rd, Tucson, AZ 85718 USA; 6 Global Patient Outcomes and Real World Evidence, Eli Lilly and Company, Lilly Corporate Center, Indianapolis, IN 46285 USA

**Keywords:** Pain, Outcome measurement, Response options, Rating scales, Response scales

## Abstract

**Background:**

The purpose of this literature review was to examine the existing patient-reported outcome measurement literature to understand the empirical evidence supporting response scale selection in pain measurement for the adult population.

**Methods:**

The search strategy involved a comprehensive, structured, literature review with multiple search objectives and search terms.

**Results:**

The searched yielded 6918 abstracts which were reviewed against study criteria for eligibility across the adult pain objective. The review included 42 review articles, consensus guidelines, expert opinion pieces, and primary research articles providing insights into optimal response scale selection for pain assessment in the adult population. Based on the extensive and varied literature on pain assessments, the adult pain studies typically use simple response scales with single-item measures of pain—a numeric rating scale, visual analog scale, or verbal rating scale. Across 42 review articles, consensus guidelines, expert opinion pieces, and primary research articles, the NRS response scale was most often recommended in these guidance documents. When reviewing the empirical basis for these recommendations, we found that the NRS had slightly superior measurement properties (e.g., reliability, validity, responsiveness) across a wide variety of contexts of use as compared to other response scales.

**Conclusions:**

Both empirical studies and review articles provide evidence that the 11-point NRS is likely the optimal response scale to evaluate pain among adult patients without cognitive impairment.

## Background

Patient-reported outcome (PRO) instruments are important for assessing the symptoms and/or impact of symptoms on patients’ lives [1–6]. Part of developing or adapting a PRO instrument includes the selection of a response scale (e.g., verbal rating scale [VRS], numeric rating scale [NRS], visual analogue scale [VAS]). Despite the importance of response scale selection for PRO instruments, there seems to be little empirical basis for the type of response scale selected, the response options, the visual orientation of the scale (i.e., vertical vs. horizontal), or the response scale verbal anchors. When considering a response scale for a PRO instrument, it is important to consider the context of use including population of interest, therapeutic area, and study implementation. Such considerations are central because response scales contribute to the precision as well as the performance of an instrument in the clinical trial setting, such as the ability to detect changes with treatment [1, 7].

Response scale selection is a critical aspect of PRO instrument development and has broad downstream implications for the usability of the measure from the patient perspective, the level of precision with which the construct of interest is measured, and the quantitative properties of the outcome score including range, standard deviation, scoring, and score interpretation guidelines, as well as the responsiveness of the measure to detect change [8]. Additional complicating factors such as placement and exact wording of response anchors, cultural comparability/translatability of the format and wording, and ability to migrate the scale to various modes of data collection (paper, electronic) should also be considered.

Pain is subjective in nature and is measured by patient report of intensity, among other subjective qualities. There is an abundance of existing literature on pain measurement, from expert opinions to consensus guidelines to empirical studies, all of which compare the suitability of response scale types within a particular context of use. In the PRO assessment field, 5- and 7-point VRS, 11-point NRS (particularly recommended for use in pain measurement but used in other areas as well [9]), and 10 cm/100 mm VAS are commonly used for adult assessments. VAS, NRS, and VRS response scales are generally reliable and valid, and are usually part of primary outcome measure(s) in clinical trials of chronic pain treatments. For these reasons, it is important to consider the empirical evidence as well as precedent, especially when considering the development of a new instrument.

Although response scales in pain measurement are well-explored, the existing literature should be further compared and analyzed for key themes and recommendations. Existing Initiative on Methods, Measurement, and Pain Assessment in Clinical Trials (IMMPACT) guidelines and review articles indicate the NRS as the gold standard in measurement of pain intensity [9, 10]; however, much of the key research was published prior to the 2009 issuance of the Food and Drug Administration’s (FDA) Guidance for Industry titled “Patient-Reported Outcome Measures: Use in Medical Product Development to Support Labeling Claims” [1]. As such, a review to determine the state of the evidence was necessary in order to guide the ongoing research at the Critical Path Institute’s PRO Consortium [11], as well as to inform the PRO assessment field during a period of expanding instrument development. The purpose of this paper is to describe a comprehensive review of the scientific literature aimed at identifying response scale options common in pain measures for the adult population, to summarize the empirical evidence in support of response scale selection, and to provide recommendations for this context of use.

## Methods

### Search procedure, terms, and strategy

The literature review was part of a larger study to summarize the available empirical evidence to support response scale selection during the development of new PRO instruments. The targeted search strategy included formal guidelines or review articles on the selection of response scales and response scale methodology (not specific to PRO instruments) and evidence on the selection of response scales for use in PRO instruments. Evidence was assembled and collated based on pre-determined categories such as selection of response scales for use in PRO instruments based on therapeutic area (e.g. pain). This paper focuses on the identification and review of literature that addressed response scale selection and use for PRO-based pain assessment in adults. To provide a comprehensive review, references included review articles, consensus guidelines, and expert opinion pieces as well as primary research studies (from the last 10 years) on response scale choice for pain assessment. The search database included Embase and MEDLINE (2004–2014; only articles published in English) and presentation abstracts from two recent annual meetings/conferences of the International Society for Pharmacoeconomics and Outcomes Research (2013–2014) and International Society for Quality of Life Research (2012–2013). The types of included references were PRO instrument articles with information on response scale selection in adults. Additional supplementary searches (e.g., reviewing reference lists of articles that met the initial search criteria) were also conducted to ensure no relevant publications were missed. These supplementary sources were not limited by publication date, and included the reference lists of key articles, publications not included in the search databases, and websites for major PRO-related working groups and consortia (e.g., PROMIS, NIH Toolbox, Medical Outcomes Study, Neuro-QoL, ASEQ-ME, EORTC, EuroQol Group, and FACIT Measurement System).

For abstracts and full-text articles, the search results were examined by two independent reviewers to determine whether the article should be included in the review; in the case of non-agreement, a third senior reviewer made the final judgement. Articles were excluded if they provided no direct or indirect evidence relevant to the search objectives, were not applicable to PRO instrument development, or addressed a therapeutic area not pre-specified for inclusion. Once articles fitting the search criteria were identified, the relevant data were extracted and summarized. The extraction tables included data on the study objective, study design, study population, therapeutic area, name of PRO instrument, type of response scale, and empirical evidence for response scale selection. To further characterize the quality of included articles, several different efforts were employed. First, included articles were categorized as providing either direct evidence or indirect evidence. Direct evidence was defined as evidence that provided a direct answer to the research question of interest. For example, direct evidence articles compared empirically the relative robustness or merits of two different response scale types within the same study/population. Indirect evidence was defined as important, relevant evidence that should be considered in the review and overall conclusions, but that does not directly answer the research question or hypothesis. Second, an assessment was made of the quality of the data presented and the strength of results and recommendations for each article included in the review. Each article was assigned a grade based on the type of article and strength of the data, from A (primary research; reflects the strongest empirical evidence for response scale recommendation) to D (review or expert opinion; reflects the weakest empirically-based evidence). In summary, the methods were comprehensive and covered several search objectives; the detailed literature review methods and processes are summarized elsewhere [12].

## Results

**Fig. 1:**
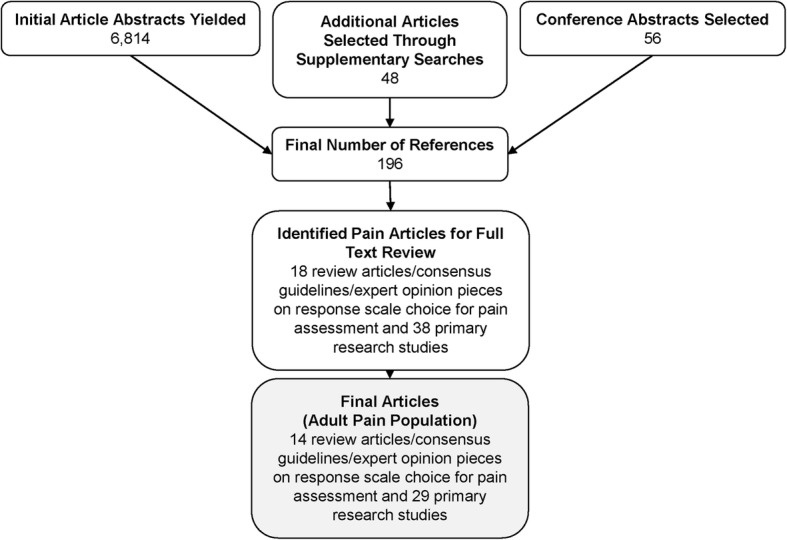
Screening and review process for pain-related references*. *The top row covers all initial seven search objectives and proceeds accordingly

Our review of the literature providing an empirical basis for response scale selection yielded pain (adult population) as the most prevalent area (Gries K, Safikhani S, Pease S, Harrington M, Rudell K, Berry P, Crescioni M, Patel M: Comprehensive literature review to characterize response scale types in patient-reported outcome measures, Unpublished observations). As a result of the abstract review, we identified 18 review articles/consensus guidelines/expert opinion pieces and 38 primary research studies for full text review, providing insights into the optimal response scale selection for pain assessment (Fig. 1). Further examination of the full text articles narrowed these down to 14 and 29, respectively.

### Response scale types used in adult pain studies

In the adult population, there were 14 review articles/consensus guidelines/expert opinion pieces and 29 primary research studies, providing insights into optimal response scale choice for pain assessment. There were seven individual response scales identified in this comprehensive review across all search objectives (i.e., VAS, VRS, NRS, Faces, other graphical, binary, and Likert or Likert-type scales). Based on review, it appears that adult pain studies have historically used simple single-item measures of pain with a VAS, NRS, or VRS as their primary outcome, albeit sometimes in the context of a multi-item instrument (e.g., the Brief Pain Inventory). There was no widely accepted standard for clinical pain assessment that would facilitate the comparison of response scale performance across trials (Table 1). Further descriptions of the response scale types are summarized elsewhere (Gries K, Safikhani S, Pease S, Harrington M, Rudell K, Berry P, Crescioni M, Patel M: Comprehensive literature review to characterize response scale types in patient-reported outcome measures, Unpublished observations).

**Table 1:** Summary of key evidence to support response scale selection in pain

Reference	Article type, evidence type ^a^, and grade ^b^	Article recommendations and rationale
Ong and Seymour 2004 [35]	Expert opinion, Direct, D	The VAS is simple, widely used, easily understood by most patients, and has strong validity. In general, the VAS is probably the most reliable and sensitive tool for measuring pain.
Dworkin et al. 2005 [9]	Consensus guideline, Direct, D	VRS and NRS are preferred over VAS by patients. VAS measures typically have greater amounts of missing data and are more difficult for older patients and patients taking opioids to understand. NRS ratings may be difficult in the presence of cognitive impairment; VRS might be easier in these circumstances. An 11-point NRS measure of pain intensity is recommended with 0 meaning “no pain” and 10 meaning “pain as bad as you can imagine.” An additional pain intensity VRS can be considered (none, mild, moderate, severe).
Williamson and Hoggart 2005 [10]	Review article, Direct, B	All three of the pain-rating scales explored in this review are reliable and valid (VAS, VRS, and NRS). As a tool for pain assessment the NRS is probably more useful than the VRS or the VAS.
Schofield et al. 2006 [36]	Review article, Direct, B	Among fifteen reviewed papers of pain tools in the adult population, one examined five different scales across a range of care home settings in the UK. It suggests that VRS was the most successful, followed by the NRS.
Litcher-Kelly et al. 2007 [37]	Review article, Direct, B	Authors researched which pain assessments were most frequently used in clinical trials. The most frequently used assessments were the single-item VAS and the NRS.
Phillips 2007 [38]	Review article, Direct, B	No recommendation provided: Tailoring the pain assessment to the individual patient and knowing when and how to use different assessment tools will substantially increase the probability of a comprehensive pain assessment. Combining the appropriate tools to identify pain intensity, location, and pain behavior is an appropriate strategy to improve assessment outcome.
Khorsan et al. 2008 [31]	Review article, Direct, B	The NRS provides intensity estimates relatively quickly, is highly patient-centered, and has the most value when looking at change within individuals. The simplest and least time-consuming measure is the NRS.
Grimmer-Somers et al. 2009 [28]	Review article, Direct, B	There is considerable detail regarding the psychometric properties of pain severity scales. VAS, VRS, and NRS are useful for quick initial assessment of one pain dimension.
Hjermstad et al. 2011 [29]	Review article, Direct, B	NRS-11, VRS-7, or VAS work well and are applicable in pain intensity assessment. These response scale types exhibit ease of use, strong psychometric properties, work well in clinical use, and/or are sensitive to symptoms.
Vela et al. 2011 [32]	Expert opinion, Direct, D	We suggest using the NRS because of its ease of use.
Cook et al. 2013 [30]	Review article, Direct, B	A 0–10 point NRS (“no pain” to “worst imaginable pain”) was chosen for the National Institutes of Health (NIH) Toolbox to measure pain intensity in adults because it is commonly used in clinical studies and has strong validity.
Riddle 2013 [39]	Review article, Direct, B	The McGill pain questionnaire and the pain VAS are the two most commonly used pain instruments in the clinical setting. Previous research has shown them to be valid and reliable in measuring acute pain.
Wolters et al. 2013 [33]	Review article, Direct, B	Our extensive review of pain intensity measures has led us to suggest that the NRS-11 be used to assess this endpoint domain in neurofibromatosis trials. It is brief, reliable, valid, and widely used. Additionally, it is highly recommended in clinical trials from pain experts and other consensus groups, is widely used in research and has strong reliability, validity, sensitivity to change, and feasibility in ages 8 years and older.
Green et al. 2014 [40]	Review article, Direct, B	The VAS-Usual pain and VAS-Worst pain were moderately correlated (*r* = 0.63), providing evidence of their structural validity.

^a^ Direct evidence: Primary research that compares different response scales within study. Indirect evidence: Review or expert opinion based on empirical evidence

^b^ Grade Key: A) Primary research; compares different response scales within the study; B) Review or expert opinion; based on an empirical evidence base; C) Primary research; evaluates a single response scale type within the study; and D) Review or expert opinion, based on expert consensus, convention or historical experience

### Summary of key evidence regarding numeric rating scales in the adult pain population

In the review of the 29 direct empirical evidence, we identified 11 articles that directly compared NRS with other type of response scales in either a validation study or a direct methodological comparison (Table 2). The following observations regarding the NRS suggest that the NRS is potentially superior to the VRS or VAS. The NRS was preferred over the VAS because it was easier to administer and score [13]; was preferred by patients [14]; was found to be more responsive [15]; and had both higher patient acceptability and better psychometric properties [16]. The NRS was found to be more responsive than a six-point VRS [17]; the most feasible and discriminative self-report scale as compared to a VAS or 5-point VRS [18]; and more sensitive and discriminative than a binary scale [19]. One study that compared an NRS to a *Faces* scale (a type of graphical scale that uses photographs or pictures showing a continuum of facial expressions) found both scales to be equally well understood [20], although a population of Swahili-speaking patients preferred the Faces scale over the NRS [21].

**Table 2:** Summary of key evidence to support the numeric rating scale (NRS) in pain

Reference	Study type, evidence type ^a^, and grade ^b^	Article recommendations and rationale
Grotle et al. 2004 [41]	Cross-sectional study, Direct, A	The NRS showed statistically higher standardized response mean SRM when compared with the VAS. The VAS format is difficult for some patients and time-consuming to score. The NRS is easier to understand and quicker to score.
Gagliese et al. 2005 [16]	Prospective study, Direct, A	The NRS should be the first choice across age groups. Compared to the other scales, it had low error rates, high face validity, and high convergent, divergent, construct and criterion validity. Results suggest that use of the VAS should be discouraged, unless practice is provided to patients.
Jackson et al. 2006 [14]	Instrument development and/or validation study, Direct, A	Most participants preferred using an NRS to the continuous VAS. Some found numbers easier to use whereas others found the scale of pain intensity (referred to by authors as the SPIN) more helpful.
Ritter et al. 2006 [13]	Cross-sectional study, Direct, A	The NRS (with graphical component) is a valid measure and was as successful as the VAS in measuring the underlying pain variable. The NRS was easier to administer and code than the VAS.
Chanques et al. 2010 [18]	Prospective study, Direct, A	The NRS should be the tool of choice for the intensive care unit (ICU) setting, because it is the most feasible and discriminative self-report scale for measuring critically ill patients’ pain intensity.
Huang et al. 2012 [21]	Instrument development and/or validation study, Direct, A	The Faces scale and NRS were adapted and translated for a population of Swahili-speaking patients in western Kenya, and demonstrated the face validity, acceptability, and field-readiness of these scales through cognitive interviewing of hospitalized patients. In this population the Faces scale was preferred over the NRS.
Van Dijk et al. 2012 [42]	Prospective study, Direct, A	A lack of agreement is found between the patients and Acute Pain Nurses on what constitutes ‘bearable’ pain in relation to the reported NRS score. These findings suggest variable interpretation of scores on an NRS.
Chien et al. 2013 [17]	Instrument development and/or validation study, Direct, A	The NRS is potentially more sensitive to clinical changes in comparison to the VRS
Huang et al. 2013 [20]	Prospective study, Direct, A	The Faces scale and the NRS were easily understood and well accepted by participants and should be implemented for daily use in the inpatient setting in order to gauge patients’ pain and response to pain treatment.
Rothaug et al. 2013 [19]	Prospective study, Direct, A	Binary answer format was proven to be a practical alternative to the NRS format for a screening instrument, however may not be as sensitive to discriminate levels of pain intensity as compared to the NRS.
Gonzalez-Fernandez et al. 2014 [15]	Cross-sectional study, Direct, A	The NRS (referred to in the article as general Labeled Magnitude Scale; essentially a VAS with the addition of numbers along the scale) has the potential to replace the VAS in the measurement of pain intensity in the clinical setting. As a minimum it may serve as an important tool in the management of patients with chronic pain.

^a^ Direct evidence: Primary research that compares different response scales within study. Indirect evidence: Review or expert opinion based on empirical evidence

^b^ Grade Key: A) Primary research; compares different response scales within the study; B) Review or expert opinion; based on an empirical evidence base; C) Primary research; evaluates a single response scale type within the study; and D) Review or expert opinion, based on expert consensus, convention or historical experience

### Summary of key evidence regarding visual analog scales in the adult pain population

Seven studies directly compared the use of the VAS with other response scales. The following observations were made to support the use of the VAS. The VAS was found to be more sensitive than a four-point VRS [22]; a VAS with intermediate verbal anchors enhanced scale comprehension compared to a traditional VAS [15]; and the VAS was found to be equally well accepted and understood as a Faces scale [23]. One study found that a four-point VRS had lower scale failure rates (i.e., one or both tests were not completed correctly), than a VAS [24]. Table 3 summarizes the key evidence regarding VAS in the assessment of pain for adults.

**Table 3:** Summary of key evidence to support the visual analog scale (VAS) in pain

Reference	Study type, evidence type ^a^, and grade ^b^	Article recommendations and rationale
Skovlund et al. 2005 [22]	Cross-sectional study, Direct, A	The VAS is consistently more sensitive than the four-point VRS.
Chan et al. 2005 [43]	Prospective study, Direct, C	The VAS as a numeric measure of pain intensity allowed for documentation of pain over time and assessment of the relationship between pain and disability and function post-hand injury.
Loos et al. 2008 [24]	Cross-sectional study, Direct, A	Because of lower scale failure rates and overlapping VAS scores per VRS category, the VRS should be favored over the VAS in future post-herniorrhaphy pain assessment.
Boomershine et al. 2011 [44]	Prospective study, Direct, C	The VAS is simple to score for longer instruments and has been shown here to be valid, sensitive to change, and able to identify with score cut offs patients with clinically significant symptoms.
Bergh et al. 2011 [45]	Clinical study, Direct, A	The VAS is more sensitive than the Pain Matchers (PM) for recording changes in pain intensity when assessing the effects of treatment on labor pain.
Dogan et al. 2012 [23]	Prospective study, Direct, A	No patients had difficulty completing either pain scale (VAS versus Faces scale) (there were illiterate patients in the sample).
Gonzalez-Fernandez et al. 2014 [15]	Cross-sectional study, Direct, A	The VAS (with some intermediate verbal anchors) has the potential to replace the VAS in the measurement of pain intensity in the clinical setting.

^a^ Direct evidence: Primary research that compares different response scales within study. Indirect evidence: Review or expert opinion based on empirical evidence

^b^ Grade Key: A) Primary research; compares different response scales within the study; B) Review or expert opinion; based on an empirical evidence base; C) Primary research; evaluates a single response scale type within the study; and D) Review or expert opinion, based on expert consensus, convention, or historical experience

### Summary of key evidence regarding verbal rating scales in the adult pain population

Three studies provided empirical evidence regarding the direct comparison of the VRS with the NRS and VAS. The following observations were made to support the use of the VRS. In 25 patients with chronic low back pain, intra-rater correlations between a VRS and VAS showed no significant difference between raters, thus demonstrating appropriate reliability [25]. A five-point VRS and VAS were administered in a cross-sectional study of patients with chronic, nociceptive, and neuropathic pain [26]. The VAS cut-off positions relative to the VRS labels and non-linear properties indicated different meaning of the rated pain intensity [26]. The VRS, NRS, and VAS were all sensitive to change for pain assessment, but the VAS was more difficult for patients in the study to understand [27]. The VRS showed substantial discrimination between pain words and was not dependent on the level of education of the patients [27]. Table 4 summarizes the key evidence regarding VRS in the assessment of pain for adults.

**Table 4:** Summary of key evidence to support the verbal rating scale (VRS) in pain

Reference	Study type, evidence type ^a^, and grade ^b^	Article recommendations and rationale
Olaogun et al. 2004 [25]	Cross-sectional study, Direct, A	The VRS and the VAS for low back pain are reliable for clinical use
Lund et al. 2005 [26]	Prospective study, Direct, A	Authors favor the VRS in pain intensity assessments over the VAS. The VAS data should be analyzed using statistical methods suitable for ordinal data.
de C. Williams et al. 2011 [27]	Prospective study, Direct, A	The VRS and NRS appeared to yield useful information in a post-conflict situation compared to the VAS which was difficult to explain and understand among participants in this study.

^a^ Direct evidence: Primary research that compares different response scales within study. Indirect evidence: Review or expert opinion based on empirical evidence

^b^ Grade Key: A) Primary research; compares different response scales within the study; B) Review or expert opinion; based on an empirical evidence base; C) Primary research; evaluates a single response scale type within the study; and D) Review or expert opinion, based on expert consensus, convention or historical experience

### Summary of results in pain

Several review articles support the usefulness, reliability, and validity of each of the scale types (i.e., the VAS, VRS, NRS) for the self-assessment of pain in adults [28, 29]. The majority of the reviewed articles, 7 out of 13, identified the NRS as the most appropriate response scale for the assessment of pain in adults, including the IMMPACT guidelines for pain measurement in clinical trials and the National Institutes of Health Toolbox pain assessment [9, 10, 30–33].

The NRS circumvents problems for translations, administration, and scoring that may occur with the VAS on paper administration by ambiguous lines being drawn. The electronic format of the VAS may now avoid some of those issues stemming from paper administration; however, potential issues remain, stemming from various formats of screen sizes, specifically that the length of the VAS line may be variable across devices.

Another consideration is that a VAS is not appropriate for telephone interview-based or interactive voice response (IVR) system-based data collection. The VAS relies on a respondent’s ability to view the VAS and mark the position on it (via a pencil/pen, touch with a stylus or finger, or a mouse click) that represents his or her answer to the specific question. Hence, the VAS is limited to paper and screen-based electronic modes of data collection that enable that to occur.

In summary, both types of articles, reviews and empirical studies, provide evidence that the 11-point NRS is likely the optimal self-report response scale to evaluate pain among adult patients without cognitive impairment. Furthermore, the 11-point NRS is the easiest to implement and utilize on electronic data collection systems that time- and date- stamp the respondent’s data entry, which is recommended within FDA’s PRO Guidance for supporting PRO-based labelling claims [1].

## Discussion

In this study, we conducted a comprehensive evaluation of the scientific literature to identify response scale option types for pain studies and to review and summarize available empirical evidence for each type of response scale by context of use, to enhance response scale selection for newly developed self-reported pain instruments in the adult population. With the publication of the IMMPACT guidelines in 2005 [9], the 11-point (i.e., 0 to 10) NRS was recommended as a core outcome measure of chronic pain treatment trials. Subsequently, the NRS became the gold standard for pain assessment in clinical trials; however, it is by no means universal. For example, some analgesics may have differential efficacy across pain types, and neuropathic versus musculoskeletal pain demonstrate different characteristics. However, extensive empirical evidence has been generated since the 2005 publication of the IMMPACT guidelines, and our review demonstrated that the recommended NRS response scale still appears to have the best performance when directly compared to other commonly used response scale types in the self-assessment of pain. NRS measures tend to be preferred over VAS measures by patients [9].VAS measures often result in more missing and incomplete data than NRS measures; this is most likely due to the easier to understand and less abstract nature of NRS measures [9]. Our findings suggested that pain assessment typically consists of single-item questions which typically use either an NRS or a VAS.

The 2014 FDA Guidance for Industry, “Analgesic Indications: Developing Drug and Biological Products” [34], provides recommendations to sponsors on the development of prescription drugs that are the subject of new drug applications for the management of acute and chronic pain as well as the management of breakthrough pain. The FDA recommends the use of a well-defined and reliable PRO instrument to assess the subject’s pain intensity. Because pain is a subjective experience, the choice of an adequate instrument to measure the primary endpoint is critical to demonstrating the efficacy of an analgesic. Therefore, it is important to consider whether a well-defined and reliable instrument already exists or can be developed for an analgesic study. It is also important that measures be based on scales or instruments that have been adequately developed for use in the population to be studied and that the instruments are appropriate for use in the setting of a clinical trial to measure change over time. The FDA’s recommendations highlight the importance of choosing an adequate response scale when selecting a pain instrument for a clinical trial.

Of course, this study has limitations. First, this review does not address the considerations for measuring the various phenotypic manifestations of pain. Pain can be categorized according to its duration, acute or chronic, as well as other characteristics, such as breakthrough pain and episodes of acute pain that occur in the context of otherwise well-controlled, chronic pain. The recommendations emerging from this review speak broadly to the concept of pain and do not reflect an examination of those intricacies due to the lack empirical evidence in the literature. In addition, this review did not discriminate between the choice of response scale for a single stand-alone item or for more than one item in a multi-dimensional pain inventory. Also, the review and merit of multi-domain PRO instruments which include pain were not included in this review. Nor does this review address the question of whether pain should be measured unidimensionally or across multiple domains.

Further, this review does not attempt to address other aspects of pain endpoints, such as recall period or worst vs. average pain intensity that may impact instrument validity. The PRO instrument’s recall period for assessing pain should be appropriate for the type of pain studied and the planned study design. The FDA recommends use of an instrument that asks the subject to assess his or her worst pain over a relatively short time period, and no longer than the past 24 h, with the assessment occurring at the same time [34].

Additionally, this review focused on adult pain and does not attempt to address other populations (e.g., cognitively impaired, pediatric), or other reporters (e.g., observer- or clinician-rated). In cases of young children or subjects who cannot provide self-report, observers (e.g., parents, caregivers) can report on observable indicators (e.g., events, behaviors, or signs) of pain (e.g., wincing, crying, or squirming). However, an observer cannot validly rate a subject’s pain intensity; and the FDA does not consider an instrument that requires an observer to do so to be well-defined or reliable [34].

Lastly, this literature review was conducted in early 2015 and was limited to articles published in English during a 10-year timespan from 2004 through 2014. Although a brief scan of the literature since 2014 did not find key evidence that would change the conclusions, the authors recognize that ending the structured review at 2014 is a limitation of this manuscript.

## Conclusions

Overall, the literature supports the NRS as the preferred scale for pain measurement; the available empirical evidence demonstrates its superior performance to other response scale types in this context of use. It is simple, straightforward, and easy to interpret. This review includes evidence from a wide range of painful conditions, including acute pain as well as chronic neuropathic and musculoskeletal indications, suggesting a relatively broad generalizability of the findings. Recommendations for future research include well-designed head-to-head comparisons of response scales used in pain measures, structured reviews of pain assessment in other populations (e.g. pediatric, geriatric), and further exploration of the measurement considerations for the various phenotypic manifestations of pain.

## Abbreviations

FDA: Food and Drug Administration
IMMPACT: Initiative on Methods, Measurement, and Pain Assessment in Clinical Trials
NRS: Numeric rating scale
PRO: Patient-reported outcome
VAS: Visual analogue scale
VRS: Verbal rating scale
